# Supplementary material for: Nutritional Provision of Iron Complexes by the Major Allergen Alt a 1 to Human Immune Cells Decreases Its Presentation
Source: Int J Mol Sci. 2023 Jul 25;24(15):11934. doi: 10.3390/ijms241511934 (PMC10418924; doi:10.3390/ijms241511934)
Supplement: Supplementary file 1 [file ijms-24-11934-s001.zip › ijms-2455438-supplementary.pdf]

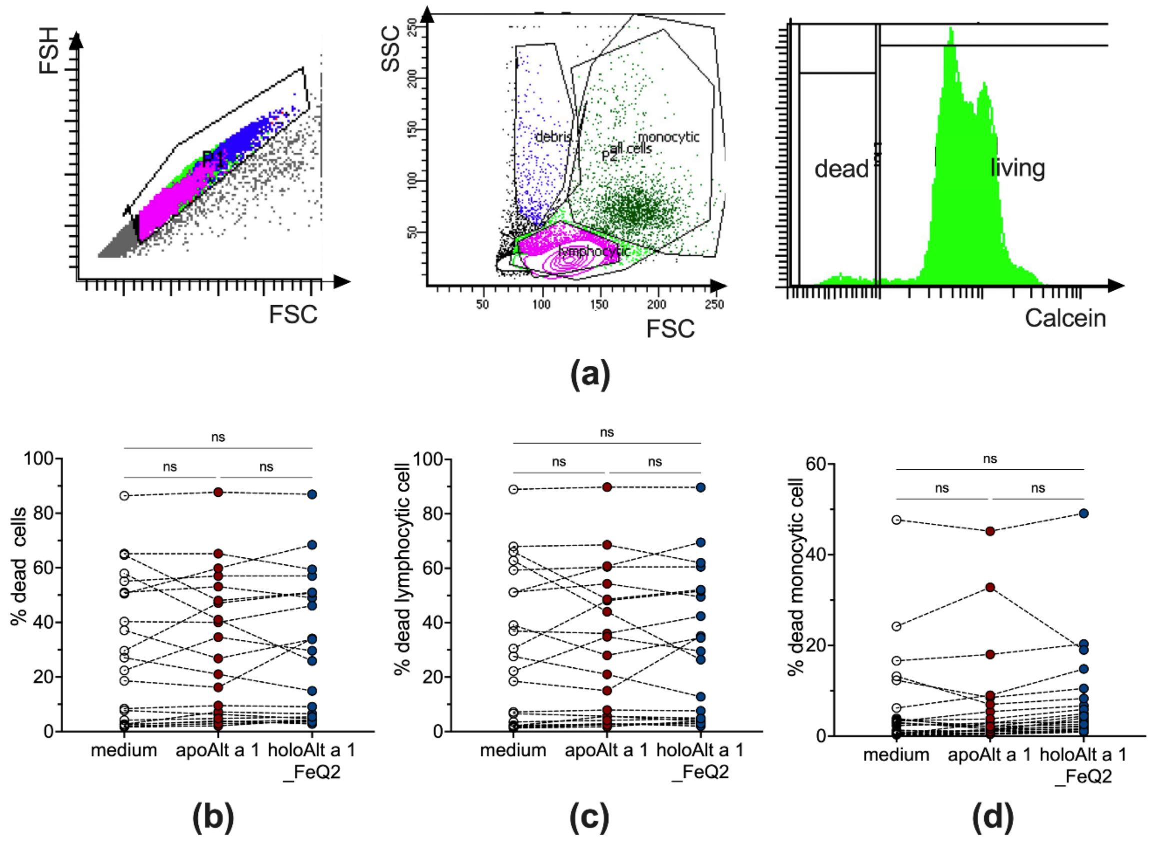

Figure S1. No significant differences in the relative numbers of dead cells in the gated cell population upon apo- or holoAlt a1 stimulation. Peripheral blood mononuclear cells were incubated overnight in iron-free media alone or in the presence apo- or holo-Alt a 1. Cells were stained with calcein-AM before flowcytometric analyses. Calcein positive cells within the gate were considered living. **(a):** Gating strategy, relative Calcein negative numbers of **(b):** all gated cells, **(c):** in the lymphocytic gate **(d):** in the monocytic gate. Groups were compared by RM-one-way ANOVA following Tukey's multiple comparisons test. n.s. not significant
